# Correlation between dietary patterns and cognitive function in older Chinese adults: A representative cross-sectional study

**DOI:** 10.3389/fnut.2023.1093456

**Published:** 2023-04-04

**Authors:** Ruoyu Gou, Jian Qin, Weiyi Pang, Jiansheng Cai, Tingyu Luo, Kailian He, Song Xiao, Xu Tang, Zhiyong Zhang, You Li

**Affiliations:** ^1^Department of Environmental Health and Occupational Medicine, School of Public Health, Guilin Medical University, Guilin, Guangxi, China; ^2^The Guangxi Key Laboratory of Environmental Exposomics and Entire Lifecycle Heath, Guilin, Guangxi, China; ^3^Department of Environmental and Occupational Health, School of Public Health, Guangxi Medical University, Nanning, Guangxi, China

**Keywords:** cognitive function, dietary patterns, older adults, vegetables, mushrooms

## Abstract

**Objective:**

The objective of this study was to investigate the relationship between dietary patterns and cognitive function in older adults (≥60 years old).

**Methods:**

Food intake was quantitatively assessed by the Food Frequency Questionnaire (FFQ), and cognitive function was assessed by the Chinese version of the Simple Mental State Examination Scale (MMSE). Four major dietary patterns were identified by the factor analysis (FA) method. The relationship between dietary patterns and cognitive function was evaluated by logistic regression.

**Results:**

A total of 884 participants were included in the study. Four dietary patterns (vegetable and mushroom, oil and salt, seafood and alcohol, and oil tea dietary patterns) were extracted. In the total population, Model III results showed that the fourth quartile of dietary pattern factor scores for the vegetable and mushroom pattern was 0.399 and 7.056. The vegetable and mushroom dietary pattern may be a protective factor for cognitive function, with *p-*value = 0.033, OR (95% CI): 0.578 (0.348, 0.951) in Model III (adjusted for covariates: sex, ethnic, marital, agricultural activities, smoking, drinking, hypertension, diabetes, dyslipidemia, BMI, and dietary fiber). In the ethnic stratification analysis, the scores of dietary pattern factors of the vegetable and mushroom among the Yao participants were 0.333 and 5.064. The Vegetable and mushroom diet pattern may be a protective factor for cognitive function, *p-value* = 0.012, OR (95% CI): 0.415 (0.206, 0.815).

**Conclusion:**

The fourth quartile of the vegetable and mushroom dietary pattern scores showed dose-dependent and a strong correlation with cognitive function. Currently, increasing vegetable and mushroom intake may be one of the effective ways to prevent and mitigate cognitive decline. It is recommended to increase the dietary intake of vegetables and mushroom foods.

## 1. Introduction

As global lifespan increases, there is a concern about diseases that significantly impact older adults, including those that cause chronic ailments, impact physical functioning, and lead to cognitive decline. Cognitive decline can have multiple causes, with Alzheimer's disease being the most common, with more than 46 million people worldwide suffering from dementia, which is impacted strongly (1, 2). It is expected that the number of Alzheimer's disease cases will increase to 131.5 million by 2050, and the aging population in China is constantly increasing. In 2020, the prevalence of cognitive decline and dementia in elderly individuals aged 60 years and above in China was 15.54% and 6.04%, respectively (3–5). Areas of cognitive dysfunction, such as progressive memory loss and language decline, are progressively impairing the ability of older adults to live independently.

The implementation of healthy dietary patterns is a potential preventive strategy for declining cognitive function and can mitigate the cognitive decline that occurs with age (6). The effects of single nutrients or foods on cognitive function have been well documented. For example, high intakes of fish, omega-3 polyunsaturated fatty acids, and high linoleic acid may reduce cognitive decline (7–9). Nutrients such as monounsaturated fatty acids (MUFAs), polyunsaturated fatty acids (PUFAs), and antioxidants (e.g., beta-carotene and vitamin C) can protect the nervous system and mitigate cognitive decline (10–12).

However, it has been found that one's entire diet should be considered and that there are synergistic effects between the nutrients consumed (stronger than their single components), meaning that dietary patterns may have a more significant impact on cognitive function (13, 14). In recent years, the focus has shifted from investigating specific nutrients or foods to generalizing dietary patterns, and some dietary patterns have become representative of the theoretical basis that the whole diet reduces cognitive decline, such as the Mediterranean, Dietary Approaches to Stop Hypertension (DASH), Mediterranean-DASH Intervention for Neurodegenerative Delay (MIND), low-fat and plant-based, traditional food, and Westernized and seafood-rich dietary patterns (15–19). The findings on dietary patterns and cognitive function vary, with plant-based dietary patterns and Mediterranean dietary patterns alleviating the decline in cognitive impairment (17, 20). These results offer hope for dietary patterns to prevent cognitive impairment. However, the greatest limitation of theoretically guided dietary patterns is that they do not provide complete access to the full information of the dietary matrix and do not reflect the true picture of dietary intake, especially for specific populations in particular regions that cannot strictly adhere to typical dietary patterns. For example, the Mediterranean dietary model does not make use of the accumulated knowledge on the relationship between dietary intake of nutrients and neurodegeneration and only considers nine food groups (13).

The most commonly used method to explore dietary patterns is factor analysis (FA) using a data-driven approach. This method is not limited by theory but reflects dietary intake in a specific population. For example, adherence to a “grain” pattern may be a risk factor for cognitive function and a “plant” pattern may be a protective factor for cognitive function (21). Gongcheng County is the “hometown of longevity” in China, and oil tea is a daily beverage for people in southwest Guangxi, among which Gongcheng oil tea (22) is the most famous. According to the analysis of China's National Food hygiene standard method, the main components of oil tea are green tea, ginger, and oil (mainly peanut oil), and its main nutritional components include tea polyphenols, gingerol, total fat, and dietary fiber. As a specialty drink of the Yao people, Chinese medicine considers oil tea to be a compound preparation of traditional Chinese medicine, which has the functions of strengthening the spleen and eliminating food, treating the common cold and loosening the bowels, relieving symptoms of diarrhea, and stopping diarrhea (22). Ginger (23) and tea polyphenols (24) have been shown to have neuroprotective effects. We found that the dietary structure of the local elderly is relatively stable and less affected by external influences. However, there are few studies on the relationship between the dietary patterns of oil tea drinkers and cognitive function. Therefore, we further investigated the relationship between dietary patterns and cognitive function in Gongcheng County to provide an epidemiological basis for the prevention of cognitive impairment in local older adults.

## 2. Participants and methods

### 2.1. Participants

The study site was Gongcheng Yao Autonomous County, Guilin, Guangxi, China. According to the cross-sectional sampling survey, the sample size calculation formula is as follows:


$$\begin{array}{l} \text{N}=\left({\text{Z}}_{\alpha }^{2}\times \text{pq}\right)/{\text{d}}^{2}, \end{array}$$

where *N* is the sample size, Z is the statistic, α = 0.05, Z = 1.96, p is the expected prevalence, q = 1-p, and d is the allowable error (d = 0.1p). The prevalence of cognitive dysfunction in rural Chinese older adults was 42.9% (25). The estimated sample size for this study was 589, with an additional 10–15% of the sample. The number of people surveyed in this cross section was *N* = 1,246. Each participant completed the MMSE and FFQ questionnaire and underwent a physical examination and laboratory tests.

Inclusion criteria were as follows: older adults who (a) resided in the study area; (b) were over 60 years of age; (c) had blood sample, height, weight, and questionnaire information; and (d) had a reasonable energy intake of 500–6000 kcal (26). Finally, the participants were propensity score matched (PSM) (1:3) by age and years of education. A total of 884 adults aged 60 years and older were included in this study (2018–2019). The study included men (*n* = 370) and women (*n* = 514).

This research was conducted in accordance with the guidelines set forth in the Declaration of Helsinki, and all procedures involving human participants were approved by the Ethics Committee of Guilin Medical College. Written informed consent was obtained from each participant (No. 20180702-3).

### 2.2. Assessment of cognitive function

The MMSE questionnaire has high reliability and validity for the assessment of cognitive function. The questionnaire has the advantage of being easily applied and implemented in a short period. To assess the relationship between dietary patterns and the risk of cognitive dysfunction, MMSE scores have been classified according to the level of literacy: illiterate group ≤17, primary group ≤20, secondary and higher group ≤24 as the cutoff point, regarded as the cognitive impairment group (CI), compared to the non-CI group, and the rest are classified as the non-cognitive impairment (non-CI) group (27, 28).

### 2.3. FA and identification of dietary patterns

The main food intake during the previous year was determined by reference to the food frequency questionnaire (FFQ), which included the frequency and quantity of food consumed (29). The intake of each group (g/day) was then calculated by adding the intake of the member's food. Daily nutrient intake was calculated by multiplying the frequency of consumption of each component of each food by the nutrient content of that particular component. In this study, foods were combined into different food groups according to their nutritional content and use. To extract dietary patterns, we divided 109 foods into 14 food groups with reference to similar nutrients: cereals and potatoes, vegetables, fruits, soybeans and nuts, poultry, gut, aquaculture, egg, milk, mushroom, alcohol, oil, salt, and oil tea. Dietary patterns were generated by utilizing the factor analysis method (FA) for 14 predefined food groups. Factor analysis is a nutritional epidemiological method. By distinguishing one or more dietary factors, we found that certain types of food tend (or not) to be ingested in the same sitting.

Feature values of >1 and scree plots were used to determine the number of dietary patterns. Furthermore, by using orthogonal transformations of rotation factors, the results were easier to interpret. Food groups with absolute factor loadings of >0.3 were significant in the dietary pattern because the food items in these food groups appeared to be strongly associated with the identified factors. Factor scores were calculated by weighting each participant's intake of the 14 food groups by factor loadings. We named dietary patterns based on food groups with factor loadings of >0.6.

### 2.4. Definition of covariates

Diabetes was defined as fasting blood glucose (FBG) of ≥7.0 mmol/L or a regimen of glucose-lowering medications (30). Hypertension was defined as systolic blood pressure (SBP) of ≥140 mmHg and/or diastolic blood pressure (DBP) of ≥90 mmHg or a regimen of antihypertensive medication (31) (for this study, hypertension includes having had hypertension and not knowing that you have hypertension but having hypertension on your test.). Dyslipidemia was defined as total cholesterol (TC) of ≥6.22 mmol/L, triacylglycerol (TG) of ≥ 2.26 mmol/L, low-density lipoprotein cholesterol (LDL-C) of ≥4.14 mmol/L as high LDL-hemorrhage, and high-density lipoprotein cholesterol (HDL-C) of <1.04 mmol/L as hypo-HDL-C (32). The following demographic information was collected through questionnaires: age (60–69, 70–79, and ≥80 years), sex (male or female), ethnicity (Han, Yao, or other), marital status (unmarried, married, or other), education (illiterate [uneducated], primary education [educated for 0–6 years], and higher education [educated for more than 6 years]), agricultural activities [involved in agriculture (plowing, planting, and weeding)], and BMI (body mass index). Participants who were retired, homemakers, unemployed for more than 1 year, or physically disabled were considered nonworking individuals, unlike those involved in physical activities in agriculture. Smoking was defined as currently smoking at least one cigarette per day. Alcohol consumption was defined as drinking at least 50 g of alcohol or more once a month (33).

### 2.5. Statistical methods

The chi-square test and Student's *t*-test were applied for continuous and categorical variables, respectively, and the Wilcoxon rank-sum test was used for comparisons between groups of nonnormally distributed information. The energy and nutrient contents of food were obtained from the Chinese Food Composition Table (2009 version) (34). Nutrients were corrected using the residual method. Variance inflation factors (VIF < 10) (35) were employed to avoid multicollinearity between nutrients. The ranges of the included nutrients are as follows: SFAs, 229.324; MUFAs, 1159.459; PUFAs, 220.699; energy, 336.380; protein, 13.121; fat, 3052.548; carbohydrates, 81.488; and dietary fiber, 2.182. Case matching and controls by age and educational years were performed using the propensity score matching method. Four dietary patterns were extracted using the factor analysis (FA) method with a factor loading matrix criterion of 0.3. Food groups that exceeded the criterion of 0.6 showed extreme correlation with their corresponding dietary patterns and were then named according to those patterns. Unconditional regression of binary logistic regression was used to fit a continuous intake of 14 food groups to the cognitive function model, and linear trend analysis was implemented. Simultaneous restricted cubic spline (RCS) plots were used to represent the relationship between continuous dietary pattern scores and cognitive function. The lowest quartile (first quartile) was defined as the reference group in each model. The results were expressed as weighted OR (95% CI). After adjusting for potential confounders, unconditional binary logistic regression was applied to analyze the relationship between dietary patterns and cognitive function and to perform between-group trend analysis stratified by ethnicity. All analyses were performed in R (4.1.3), and two-tailed *p*-values of <0.05 were considered statistically significant. The authors can be contacted for the source code should there be any questions about the analysis.

## 3. Result

### 3.1. Participant characteristics

The propensity score matching (PSM) results were as follows: non-CI (non-cognitive impairment) (*N* = 663) and CI (cognitive impairment group, compared to the non-CI group.) (*N* = 221). The jitter scatter plot and the histogram of the propensity value distribution show good results (Supplementary Figure 1).

Statistically significant differences occurred between the sex (*P-*values = 0.001), hypertension (*P-*values = 0.049), MMSE scores, orientation, memory, attention calculation, language skills, and visuospatial skills (*P-*values < 0.001) of the non-CI and CI groups, as shown in Table 1. Different ethnic groups had statistically significant variations in sociodemographic characteristics, including marital status (*P-*values = 0.009) and agricultural activities (*P-*values = 0.009), as shown in Table 2.

**Table 1:** Sociodemographic characteristics of participants.

**Characteristics**	**All participants (*n* = 884)**	**Non-CI (*n* = 663)**	**CI (*n* = 221)**	***P*-value**
**Age, (years)** ^a^				
60–69	662 (74.89)	500 (75.41)	162 (73.30)	0.547
70–79	185 (20.93)	138 (20.81)	47 (21.27)	
≥80	37 (4.19)	25 (3.77)	12 (5.43)	
**Sex**				
Male	370 (41.86)	299 (45.10)	71 (32.13)	0.001
Female	514 (58.14)	364 (54.90)	150 (67.87)	
**Ethnic**				
Han	321 (36.31)	249 (37.56)	72 (32.58)	0.254
Yao	537 (60.75)	397 (59.88)	140 (63.35)	
Other	26 (2.94)	17 (2.56)	9 (4.07)	
**Marital**				
Unmarried	23 (2.60)	18 (2.71)	5 (2.26)	0.098
married	652 (73.76)	500 (75.41)	152 (68.78)	
Other	209 (23.64)	145 (21.87)	64 (28.96)	
**Education**				
Illiteracy	530 (59.95)	402 (60.63)	128 (57.92)	0.743
Primary	130 (14.71)	97 (14.63)	33 (14.93)	
Above education	224 (25.34)	164 (24.74)	60 (27.15)	
**Agricultural activities**				
No	50 (5.66)	42 (6.33)	8 (3.62)	0.179
Yes	834 (94.34)	621 (93.67)	213 (96.38)	
**Smoking**				
Yes	181 (20.48)	142 (21.42)	39 (17.65)	0.268
No	703 (79.52)	521 (78.58)	182 (82.35)	
**Alcohol drinking**				
Yes	307 (34.73)	234 (35.29)	73 (33.03)	0.596
No	577 (65.27)	429 (64.71)	148 (66.97)	
**Hypertension**				
Yes	557 (63.01)	405 (61.09)	152 (68.78)	0.049
No	327 (36.99)	258 (38.91)	69 (31.22)	
**Diabetes**				
Yes	80 (9.05)	58 (8.75)	22 (9.95)	0.685
No	804 (90.95)	605 (91.25)	199 (90.05)	
Age, (years)^b^	67.59 (5.53)	67.48 (5.43)	67.92 (5.84)	0.312
MMSE SCORE	22.97 (5.29)	25.11 (3.41)	16.54 (4.67)	< 0.001
BMI	22.52 (7.38)	22.63 (8.34)	22.17 (2.95)	0.422
Orientational^c^	9.00 [7.00, 10.00]	9.00 [8.00, 10.00]	6.00 [4.00, 8.00]	< 0.001
Memory	5.00 [3.00, 6.00]	5.00 [4.00, 6.00]	3.00 [2.00, 4.00]	< 0.001
Attention calculation	3.00 [1.00, 5.00]	4.00 [1.00, 5.00]	1.00 [0.00, 1.00]	< 0.001
Language	8.00 [7.00, 8.00]	8.00 [7.00, 8.00]	7.00 [6.00, 8.00]	< 0.001
Visual Space	0.00 [0.00, 1.00]	1.00 [0.00, 1.00]	0.00 [0.00, 0.00]	< 0.001

a *n* (%), chi-square.

b [mean (SD)], Students' *t*-test.

c Mean [25, 75], Wilcoxon rank-sum test.

**Table 2:** Sociodemographic characteristics of participants.

**Characteristics**	**Non-CI**			**CI**		
	**Han (*****n*** = **249)**	**Yao (*****n*** = **397)**	***P*** **-value**	**Han (*****n*** = **72)**	**Yao (*****n*** = **140)**	***P-*** **value**
**Age, (years)**						
60–69	183 (73.49)	301 (75.82)	0.210	49 (68.06)	105 (75.00)	0.606
70–79	59 (23.69)	78 (19.65)		19 (26.39)	27 (19.29)	
≥80	7 (2.81)	18 (4.53)		4 (5.56)	8 (5.71)	
**Sex**						
Male	102 (40.96)	191 (48.11)	0.147	20 (27.78)	48 (34.29)	0.628
Female	147 (59.04)	206 (51.89)		52 (72.22)	92 (65.71)	
**Marital**						
Unmarried	12 (4.82)	6 (1.51)	0.071	0 (0.00)	4 (2.86)	0.009
married	177 (71.08)	309 (77.83)		45 (62.50)	104 (74.29)	
Other	60 (24.10)	82 (20.65)		27 (37.50)	32 (22.86)	
**Education**						
Illiteracy	166 (66.67)	226 (56.93)	0.170	44 (61.11)	77 (55.00)	0.069
Primary	30 (12.05)	65 (16.37)		5 (6.94)	28 (20.00)	
Above education	53 (21.29)	106 (26.70)		23 (31.94)	35 (25.00)	
**Agricultural activities**						
No	12 (4.82)	27 (6.80)	0.092	6 (8.33)	1 (0.71)	0.009
Yes	237 (95.18)	370 (93.20)		66 (91.67)	139 (99.29)	
**Smoking**						
Yes	46 (18.47)	91 (22.92)	0.292	9 (12.50)	29 (20.71)	0.289
No	203 (81.53)	306 (77.08)		63 (87.50)	111 (79.29)	
**Alcohol drinking**						
Yes	85 (34.14)	143 (36.02)	0.888	20 (27.78)	50 (35.71)	0.508
No	164 (65.86)	254 (63.98)		52 (72.22)	90 (64.29)	
**Hypertension**						
Yes	163 (65.46)	235 (59.19)	0.066	51 (70.83)	94 (67.14)	0.721
No	86 (34.54)	162 (40.81)		21 (29.17)	46 (32.86)	
**Diabetes**						
Yes	20 (8.03)	37 (9.32)	0.780	8 (11.11)	12 (8.57)	0.383
No	229 (91.97)	360 (90.68)		64 (88.89)	128 (91.43)	
Age	68.09 (5.11)	67.23 (5.64)	0.013	68.94 (5.95)	67.52 (5.79)	0.138
MMSE SCORE	24.77 (3.48)	25.30 (3.35)	0.114	16.96 (4.49)	16.61 (4.41)	0.010
BMI	22.18 (3.12)	22.89 (10.45)	0.550	22.43 (2.74)	22.03 (3.06)	0.647
Orientational	9.00 [8.00, 10.00]	9.00 [8.00, 10.00]	0.633	6.00 [3.75, 8.00]	6.00 [4.00, 7.00]	0.212
Memory	5.00 [4.00, 6.00]	5.00 [4.00, 6.00]	0.084	3.00 [1.75, 4.00]	3.00 [2.00, 4.00]	0.088
Attention calculation	4.00 [1.00, 5.00]	4.00 [2.00, 5.00]	0.400	1.00 [0.00, 2.00]	1.00 [0.00, 1.00]	0.959
Language	8.00 [7.00, 8.00]	8.00 [8.00, 8.00]	0.869	7.00 [6.00, 8.00]	7.00 [7.00, 8.00]	0.303
Visual Space	0.00 [0.00, 1.00]	1.00 [0.00, 1.00]	0.245	0.00 [0.00, 0.00]	0.00 [0.00, 0.25]	0.964

Significant differences were observed in the dietary pattern scores of SFAs (*P-*values = 0.040), protein (*P-*values = 0.010), carbohydrate (*P-*values = 0.027), dietary fiber (*P-*values = 0.025), fruit (*P-*values = 0.008), soybeans and nuts (*P-*values = 0.037), poultry (*P-*values = 0.030), aquaculture (*P-*values = 0.005), eggs (*P*-values < 0.001), mushrooms (*P*-values < 0.001), and vegetables and mushrooms (*P-*values = 0.001) of the non-CI and CI groups, as shown in Table 3. For the non-CI and CI groups, dietary pattern scores for PUFAs (*P-*values = 0.008), soybeans and nuts (P *P-*values = 0.002), oil tea (P *P-*values = 0.020), oil and salt (*P-*values = 0.042), and seafood and alcohol (*P-*values = 0.049) significantly varied among different ethnic groups, as shown in Table 4.

**Table 3:** Nutrients and factor scores of participants with cognitive impairment and normal group.

**Characteristics**	**All participants (*N* = 884)**	**Non-CI (*N* = 663)**	**CI (*N* = 221)**	***P*-value**
SFAs, (g)	15.93 [9.70, 24.30]	16.25 [10.18, 24.80]	14.61 [9.02, 22.90]	0.040
MUFA, (g)	23.56 [15.33, 35.56]	23.89 [15.47, 35.80]	22.05 [15.20, 34.88]	0.242
PUFA, (g)	12.93 [7.56, 21.03]	12.79 [7.64, 20.35]	13.24 [7.46, 22.16]	0.417
Energy, (kcal)	1382.30 [1014.15, 1848.75]	1401.30 [1029.37, 1871.07]	1278.63 [948.68, 1721.94]	0.041
Protein, (g)	33.66 [22.38, 48.63]	34.47 [23.29, 50.36]	32.01 [19.84, 43.07]	0.010
Fat, (g)	57.64 [39.54, 88.76]	58.57 [39.66, 88.76]	54.62 [38.91, 88.28]	0.299
Carbohydrates, (g)	171.02 [123.26, 225.95]	173.81 [126.23, 230.37]	164.88 [115.47, 215.53]	0.027
Dietary fiber, (g)	12.75 [9.68, 16.60]	12.98 [9.94, 16.91]	11.63 [9.05, 15.86]	0.025
Cereals and potatoes, (g/day)	519.38 [362.80, 763.26]	519.55 [365.58, 757.31]	518.29 [355.05, 776.62]	0.460
Vegetables, (g/day)	212.66 [114.88, 393.45]	217.01 [117.80, 394.58]	210.16 [105.27, 376.70]	0.254
Fruits, (g/day)	123.90 [54.73, 275.35]	126.53 [58.47, 287.20]	114.59 [34.32, 211.63]	0.008
Soybeans and nuts, (g/day)	16.06 [5.84, 35.12]	16.91 [6.23, 35.67]	11.73 [4.86, 30.84]	0.037
Poultry, (g/day)	46.27 [18.55, 93.15]	50.00 [19.94, 96.38]	36.70 [16.06, 81.18]	0.030
Gut, (g/day)	0.00 [0.00, 1.68]	0.00 [0.00, 1.68]	0.00 [0.00, 1.68]	0.360
Aquaculture, (g/day)	6.70 [2.57, 16.75]	6.70 [3.35, 16.94]	5.03 [0.00, 13.40]	0.005
Egg, (g/day)	15.84 [4.42, 50.00]	20.11 [7.24, 50.00]	11.56 [3.62, 37.00]	< 0.001
Milk, (g/day)	0.00 [0.00, 21.53]	0.00 [0.00, 23.45]	0.00 [0.00, 16.75]	0.284
Mushrooms, (g/day)	1.34 [0.00, 3.36]	1.68 [0.00, 3.36]	0.16 [0.00, 2.35]	< 0.001
Alcohol, (g/day)	0.00 [0.00, 25.12]	0.00 [0.00, 25.00]	0.00 [0.00, 26.68]	0.499
oil, (g/day)	25.80 [16.00, 41.55]	25.80 [15.90, 42.00]	25.80 [16.50, 40.50]	0.733
Salt, (g/day)	6.90 [4.50, 10.80]	6.90 [4.50, 10.50]	6.90 [4.80, 12.00]	0.383
Oil tea, (g/day)	360.00 [180.00, 640.00]	360.00 [180.00, 640.00]	360.00 [180.00, 640.00]	0.447
Vegetables and mushrooms dietary patterns score	−0.30 [−0.69, 0.40]	−0.25 [−0.66, 0.46]	−0.42 [−0.74, 0.15]	0.001
Oil and salt dietary patterns score	−0.16 [−0.56, 0.34]	−0.16 [−0.58, 0.32]	−0.16 [−0.55, 0.43]	0.446
Seafood and alcohol dietary patterns score	−0.24 [−0.46, 0.19]	−0.23 [−0.45, 0.21]	−0.26 [−0.48, 0.13]	0.147
Oil tea dietary patterns score	−0.09 [−0.52, 0.46]	−0.09 [−0.49, 0.49]	−0.09 [−0.55, 0.35]	0.253

Mean [25, 75], Wilcoxon rank-sum test.

**Table 4:** Nutrients in the cognitively impaired and normal group, and factor scores by ethnicity.

**Characteristics**	**Non-CI**			**CI**		***P*-value**
	**Han (*****n*** = **249)**	**Yao (*****n*** = **397)**	***P*** **-value**	**Han (*****n*** = **72)**	**Yao (*****n*** = **140)**	
SFAs, (g)	16.52 [10.09, 26.25]	15.85 [10.42, 23.60]	0.190	12.96 [8.83, 20.79]	16.51 [9.19, 24.12]	0.361
MUFA, (g)	24.16 [15.11, 36.37]	23.45 [15.79, 35.32]	0.082	20.41 [14.87, 30.35]	24.95 [15.92, 39.02]	0.173
PUFA, (g)	12.17 [7.01, 19.49]	12.87 [7.67, 20.32]	0.008	11.05 [6.78, 17.20]	14.48 [7.77, 24.41]	0.093
Energy, (kcal)	1339.24 [976.07, 1894.25]	1412.47 [1067.05, 1850.93]	0.155	1246.82 [925.87, 1629.49]	1312.30 [1026.62, 1922.56]	0.644
Protein, (g)	33.77 [22.61, 49.62]	34.83 [23.57, 50.40]	0.251	32.37 [21.14, 42.84]	31.78 [18.87, 45.39]	0.551
Fat, (g)	57.20 [38.45, 85.71]	58.87 [40.65, 88.59]	0.061	51.16 [36.08, 74.20]	61.09 [40.42, 98.76]	0.169
Carbohydrates, (g)	166.96 [128.86, 219.32]	181.25 [123.95, 234.53]	0.378	154.33 [121.20, 215.59]	165.60 [116.52, 214.76]	0.882
Dietary fiber, (g)	12.90 [9.90, 16.23]	13.01 [9.96, 17.08]	0.128	11.13 [8.52, 14.53]	12.25 [9.90, 16.28]	0.093
Cereals and potatoes, (g/day)	519.50 [377.97, 710.70]	519.55 [362.90, 768.70]	0.976	582.00 [344.97, 880.20]	490.84 [359.11, 722.39]	0.522
Vegetables, (g/day)	203.09 [113.72, 378.10]	217.35 [124.71, 398.77]	0.391	206.78 [118.39, 325.82]	210.51 [104.50, 407.10]	0.942
Fruits, (g/day)	120.86 [58.10, 269.76]	128.83 [59.90, 286.69]	0.567	120.87 [32.96, 204.61]	112.67 [44.89, 236.90]	0.315
Soybeans and nuts, (g/day)	12.37 [4.16, 32.30]	18.75 [8.24, 36.88]	0.002	13.36 [5.29, 29.57]	10.78 [4.85, 32.96]	0.402
Poultry, (g/day)	49.53 [19.04, 100.99]	49.50 [20.94, 93.64]	0.13	36.08 [12.61, 68.02]	37.44 [16.80, 91.26]	0.541
Gut, (g/day)	0.00 [0.00, 1.34]	0.00 [0.00, 1.68]	0.5	0.00 [0.00, 0.00]	0.00 [0.00, 2.05]	0.301
Aquaculture, (g/day)	6.70 [3.35, 16.75]	6.70 [3.35, 16.75]	0.766	5.03 [0.00, 10.21]	5.35 [0.00, 15.32]	0.288
Egg, (g/day)	22.26 [6.42, 51.88]	18.53 [7.64, 50.00]	0.798	11.56 [3.62, 40.62]	11.56 [3.35, 33.31]	0.169
Milk, (g/day)	0.00 [0.00, 23.45]	0.00 [0.00, 21.44]	0.897	0.00 [0.00, 16.75]	0.00 [0.00, 16.75]	0.937
Mushrooms, (g/day)	0.84 [0.00, 3.35]	1.68 [0.00, 4.02]	0.067	0.00 [0.00, 1.79]	0.60 [0.00, 3.35]	0.526
Alcohol, (g/day)	0.00 [0.00, 12.40]	0.00 [0.00, 44.22]	0.462	0.00 [0.00, 10.91]	0.00 [0.00, 37.11]	0.469
oil, (g/day)	25.80 [16.80, 42.00]	25.20 [15.00, 40.80]	0.369	24.90 [16.42, 39.90]	26.85 [16.42, 45.00]	0.504
Salt, (g/day)	6.60 [4.20, 9.60]	7.50 [4.80, 10.80]	0.085	6.00 [4.72, 9.90]	7.35 [5.10, 12.30]	0.100
Oil tea, (g/day)	400.00 [160.00, 640.00]	360.00 [180.00, 640.00]	0.343	440.00 [175.00, 740.00]	360.00 [180.00, 640.00]	0.020
Vegetables and mushrooms dietary patterns score	−0.44 [−0.75, 0.06]	−0.75 [−0.85, −0.31]	0.184	−0.44 [−0.75, 0.06]	−0.75 [−0.85, −0.31]	0.184
Oil and salt dietary patterns score	−0.11 [−0.51, 0.59]	−0.20 [−0.49, 0.47]	0.042	−0.11 [−0.51, 0.59]	−0.20 [−0.49, 0.47]	0.042
Seafood and alcohol dietary patterns score	−0.23 [−0.43, 0.18]	−0.13 [−0.39, −0.06]	0.049	−0.23 [−0.43, 0.18]	−0.13 [−0.39, −0.06]	0.049
Oil tea dietary patterns score	−0.07 [−0.53, 0.22]	−0.25 [−0.90, −0.13]	0.216	−0.07 [−0.53, 0.22]	−0.25 [−0.90, −0.13]	0.216

### 3.2. Dietary patterns

Kaiser–Meyer–Olkin (KMO) factor adequacy is equal to 0.81, Bartlett's test of sphericity is less than 0.001, and the cumulative variance explained is 44.64%, all of which meet the conditions for the use of factor analysis. The left side of the Scree plots shows the characteristic roots, and the slope for 4–5 factors becomes flatter, with the first four factors already covering most of the information, as shown in Supplementary Figure 2. Thus, four can be chosen as the number of factors. The dietary patterns were named according to the factor loadings of the 14 food groups in the dietary patterns (vegetables and mushrooms, oil, and salt, seafood and alcohol, and oil tea), as shown in Table 5. The first factor, which was defined by a high intake of vegetables, mushrooms, fruits, cereal potatoes, soybean nuts, gut, egg, and milk, was labeled “vegetables and mushroom dietary pattern.” The second factor, which was defined by a high intake of oil, salt, poultry, and soybean nuts, was labeled the “oil and salt dietary pattern.” The third factor, which was defined by a high intake of aquaculture, alcohol, poultry, and fruits, was labeled “seafood and alcohol dietary pattern.” The fourth factor, which was defined by a high intake of oil tea, milk, and gut, was labeled the “oil tea dietary pattern.”

**Table 5:** Factor loadings of four principal components of 14 food groups extracted from principal component analysis (PCA) of eating frequency data of 884 adults aged 60 years or older.

**Food groups**	**Vegetables and mushrooms dietary patterns**	**Oil and salt dietary patterns**	**Seafood and alcohol dietary patterns**	**Oil tea dietary patterns**
Cereals potatoes	0.462	−0.101	0.083	0.23
Vegetables	**0.604**	0.193	0.186	0.006
Fruits	0.581	−0.004	0.362	−0.071
Soybeans nuts	0.564	0.408	0.034	0.08
Poultry	0.249	0.389	0.378	0.272
Gut	0.334	0.0616	0.082	0.518
Aquaculture	0.112	0.082	**0.734**	0.017
Egg	0.553	0.073	−0.057	−0.048
Milk	0.479	−0.172	−0.094	−0.489
Mushrooms	**0.602**	0.006	−0.008	0.225
Alcohol	−0.015	−0.102	**0.697**	−0.043
oil	0.059	**0.696**	−0.042	−0.104
Salt	−0.001	**0.703**	0.007	−0.008
Oil tea	0.028	−0.163	−0.112	**0.644**
Factor variance	2.323	1.426	1.374	1.127
Variance contribution	16.59%	10.19%	9.82%	8.05%
Cumulative variance contribution	16.59%	26.78%	36.59%	44.64%

Food groups with factor loadings: food groups ≥0.600 are marked in bold.

### 3.3. Linear trend of 14 food groups and cognitive function scores

There was a nonlinear relationship between consumption and cognitive function values in the cereal and potato, fruit, and poultry food groups, with *P*-values of *p* = 0.009, *p* = 0.01, and *p* = 0.024, respectively (Supplementary Figure 3-1). There was also a non-linear relationship between dietary intake and cognitive function in the aquaculture, egg, milk, and mushroom food groups, with *P*-values of *p* = 0.001, *p* < 0.001, *p* = 0.037, and *p* = 0.045, respectively (Supplementary Figure 3-2).

### 3.4. Linear trend of factor scores of dietary patterns and cognitive function

We used restricted cubic spline (RCS) to flexibly model and visualize the relationship between dietary pattern scores and cognitive function. The seafood and alcohol dietary pattern showed a non-linear relationship with cognitive function, with a trend consisting of an initial decline, a brief increase, and a final flattening off, *P-*values = 0.003 (Supplementary Figure 4).

### 3.5. Association of dietary patterns with the risk of cognitive function

In Model I, after adjusting for sex and ethnicity, the score of the vegetable and mushroom dietary pattern factor was in the fourth quartile (0.399, 7.056); the vegetable and mushroom dietary pattern may be a protective factor for cognitive function, with an OR (95% CI) of 0.484 (0.302, 0.769). Other dietary patterns were not associated with cognitive function. In Model II, after adjusting for sex, ethnicity, marital status, agricultural activities, smoking, alcohol consumption, hypertension, diabetes, dyslipidemia, and BMI, when the score of the vegetable and mushroom dietary pattern factor was in the fourth quartile (0.399 and 7.056), it was found that the vegetable and mushroom dietary pattern may be a protective factor of cognitive function, with an OR (95% CI) of 0.517 (0.320, 0.830). Other dietary patterns were not associated with cognitive function. In Model III, after adjusting for sex, ethnicity, marital status, agricultural activities, smoking, alcohol consumption, hypertension, diabetes, dyslipidemia, BMI, and dietary fiber, when the score of the vegetable and mushroom dietary pattern factor was in the fourth quartile (0.399, 7.056), it was found that the vegetable and mushroom dietary pattern may be a protective factor of cognitive function, with an OR (95% CI) of 0.578 (0.348, 0.951). Other dietary patterns were not associated with cognitive function. A linear trend was noted between the quartiles of the dietary scores of the vegetable and mushroom (*P-*values = 0.017) and oil tea (*P-*values = 0.042) dietary patterns, as shown in Table 6.

**Table 6:** Different potential confounders entered the logistic regression model and participants adhered to the relationship between different dietary patterns and cognitive functioning.

**Subgroups**		**Model I** ^**a**^		**Model II** ^**b**^		**Model III** ^**c**^		***P*** **for trend**
		***P*** **-value**	**OR (95% CI)**	***P*** **-value**	**OR (95% CI)**	***P*** **-value**	**OR (95% CI)**	
**Vegetable and mushroom dietary pattern**								
[−2.458, −0.686]	(*N* = 221)	ref	ref	ref	ref	ref	ref	0.017
(−0.686, −0.304]	(*N* = 221)	0.216	0.763 (0.496, 1.170)	0.244	0.772 (0.499, 1.191)	0.256	0.777 (0.503, 1.199)	
(−0.304, 0.399]	(*N* = 221)	0.086	0.683 (0.441,1.053)	0.158	0.670 (0.425, 1.050)	0.236	0.762 (0.484,1.194)	
(0.399, 7.056]	(*N* = 221)	0.002	0.484 (0.302, 0.769)	0.007	0.517 (0.320, 0.830)	0.033	0.578 (0.348, 0.951)	
**Oil and salt dietary pattern**								
[−2.3456, −0.565]	(*N* = 221)	ref	ref	ref	ref	ref	ref	0.713
(−0.565, −0.163]	(*N* = 221)	0.755	1.074 (0.687, 1.681)	0.781	1.066 (0.678, 1.680)	0.837	1.049 (0.666, 1.654)	
(−0.163, 0.344]	(*N* = 221)	0.885	0.967 (0.611, 1.530)	0.911	0.974 (0.611, 1.552)	0.801	0.942 (0.589, 1.505)	
(0.344, 11.417]	(*N* = 221)	0.457	1.186 (0.757, 1.862)	0.433	1.199 (0.762, 1.893)	0.55	1.150 (0.727, 1.824)	
**Seafood and alcohol dietary pattern**								
[−1.866, −0.456]	(*N* = 222)	ref	ref	ref	ref	ref	ref	0.444
(-0.456, −0.237]	(*N* = 219)	0.464	0.843 (0.534, 1.330)	0.481	0.848 (0.534, 1.342)	0.541	0.866 (0.545, 1.373)	
(−0.237, 0.192]	(*N* = 222)	0.863	1.039 (0.671, 1.610)	0.797	1.060 (0.679, 1.655)	0.737	1.079 (0.691, 1.687)	
(0.192, 14.337]	(*N* = 221)	0.814	0.944 (0.585, 1.521)	0.675	0.898 (0.542, 1.482)	0.714	0.910 (0.550, 1.503)	
**Oil tea dietary pattern**								
[−4.010, −0.515]	(*N* = 222)	ref	ref	ref	ref	ref	ref	0.042
(−0.515, −0.086]	(*N* = 220)	0.055	0.640 (0.405, 1.006)	0.052	0.634 (0.399, 1.002)	0.057	0.639 (0.401, 1.010)	
(−0.086, 0.46]	(*N* = 221)	0.929	1.020 (0.664, 1.565)	0.947	1.015 (0.658, 1.566	0.977	1.006 (0.652, 1.553)	
(0.46, 9.162]	(*N* = 221)	0.176	0.729 (0.459, 1.150)	0.13	0.700 (0.439, 1.108)	0.101	0.677 (0.424, 1.076)	

a This logistic regression is adjusted by the following potential confounding factors: sex and ethnic.

b This logistic regression is adjusted by the following potential confounding factors: sex, ethnic, marital, agricultural activities, smoking, drinking, hypertension, diabetes, dyslipidemia, and BMI.

c This logistic regression is adjusted by the following potential confounding factors: sex, ethnic, marital, agricultural activities, smoking, drinking, hypertension, diabetes, dyslipidemia, BMI, and dietary fiber.

## 4. Discussion

This research was a case–control study based on a cross-sectional survey (*N* = 884). In a study of elderly people in Gongcheng County, female participants had a higher prevalence of cognitive impairment than other rural Chinese populations. There is substantial variation when considering the beneficial effects of diet in studies of the association of diet and cognitive impairment with age, and one potential reason for this variation in dietary pattern-cognitive function association may be sex (36, 37). Female participants should give greater attention to dietary intake. Our study points to a higher rate of cognitive impairment in hypertensive patients than in participants without cognitive impairment. Previous studies have suggested that hypertension is a risk factor for cognitive decline (38), and this association is mediated through microvascular brain damage (39). Appropriate blood pressure management may help alleviate cognitive decline in participants.

Four dietary patterns (vegetables and mushroom, oil and salt, seafood and alcohol, and oil tea) were extracted. The vegetable and mushroom dietary pattern (a) showed significant outcomes when the dietary pattern scores were at the fourth quartile after different levels of adjustment for potential confounders and (b) may be a protective factor for cognitive function. Among the Yao respondents, the vegetable and mushroom dietary pattern showed strong correlations with cognitive health when the dietary pattern scores were at the fourth quartile and after adjusting for different degrees of potential confounding factors. It is suggested that vegetable and mushroom dietary pattern may be a protective factor for cognitive function. After reviewing the previous literature, we made even more surprising findings. The Mediterranean dietary pattern includes various vegetables and mushrooms. Strict adherence to the Mediterranean diet (MD) may result in better cognitive status (40, 41). In a cross-sectional and longitudinal study of older adults in Taiwan, a dietary pattern high in phytonutrient-rich plant foods (fruits, whole grains, nuts/seeds, and vegetables) was associated with better cognitive function in older adults (42). A large epidemiological study in rural Shanxi, China, reported that two dietary patterns, the MVF (mushroom, vegetable, and fruit) and the MS (meat and soybean products) patterns, were predominant in the area, with greater adherence to the former being a possible protective factor for cognitive function (43). In the Chinese Longitudinal Healthy Longevity Survey (CLHLS), which assesses cognitive function using the Chinese version of the MMSE, a lower intake of fresh fruits and vegetables was significantly associated with a higher risk of cognitive impairment (44). Dietary intake patterns dominated by vegetables and mushrooms appear to have a protective effect on cognitive function, an outcome that is consistent with our findings. The likely reason for this protective effect is that antioxidants in the brain protect brain tissue from damage caused by free radicals, and eating more antioxidant-rich vegetables and fruits is strongly linked to the mitigation of damage in Alzheimer's patients (45). More prospective studies are needed to explore the correlation between diet and cognitive function and to determine the underlying molecular mechanisms. However, other studies indicate that cognitive function is not associated with the vegetable and mushroom dietary pattern. As discussed, the Mediterranean dietary pattern is known to include an abundance of vegetables and mushrooms. A New Zealand cohort study of older adults reported no significant associations between the Mediterranean dietary pattern and cognitive function or components of cognitive function (16). A study in Taiwan noted that a) in adults aged 65–74 years, a Western diet was detrimental to the maintenance of cognitive function, and b) in adults aged ≥75 years, the dietary pattern may not be correlated with cognitive function (46). The reason for those outcomes may be as follows: first, the Taiwan study adjusted for lifestyle factors and ADL, but no adjustment was made for nutrients, BMI, common diseases (including hypertension, diabetes, and dyslipidemia), and the covariate adjustment effect on the relationship between dietary pattern and cognitive function. Second, the healthy diet pattern involved a small sample size, thereby weakening the research strength. Finally, obvious differences in eating habits occur between rural and urban populations.

Oil and salt were not correlated with cognitive function. This finding is inconsistent with previous studies, which suggest that the oil and salt dietary pattern may be a risk factor for cognitive function (47). Dietary salt promotes neurovascular and cognitive dysfunction through gut-initiated TH17 participants (48), and high salt intake is associated with an increased risk of hypertension (49). Hypertension is an important risk factor for cognitive impairment (38), resulting in a small sample size that may lead to reduced statistical power for oil and salt dietary patterns to correlate with cognitive function.

The seafood and alcohol dietary pattern may not be associated with cognitive function. Previous reports suggest that the dietary patterns of older adult French people may be beneficial for cognitive function (fish for males and fruits and vegetables for females) (50). However, alcohol was not a protective factor for cognitive function (51). The complexity of this pattern could explain, to some extent, this inconsistent finding. Furthermore, the reason for the lack of association between the seafood and alcohol dietary pattern and cognitive function may be due to reverse causality. People with cognitive impairment may be advised to change their dietary habits and food choices. In conclusion, these possibilities cannot be ruled out in our analysis.

Oil tea dietary patterns were not associated with decreased cognitive function. Previous studies have reported that high doses of oil tea intake may be associated with a low risk of abnormal HDL cholesterol (29). This study explored the relationship between oil tea intake and blood lipids by grouping oil tea intake doses into more detailed quartiles. Recent studies have suggested that the weekly dietary intake of oil tea may be associated with diabetes under different dietary patterns (22). It may be implied that more detailed investigations should be conducted to explore the relationship between oil tea dietary patterns and cognitive function.

### 4.1. Limitations

This study also has some limitations that must be carefully considered. First, this study is a cross-sectional study, consisting mainly of older Chinese adults, and therefore, it is not possible to establish a clear chronological order or to exclude the possibility of reverse causality and the existence of Neyman bias. Second, the dietary intake assessment was not performed before the cognitive function measures, and we cannot infer the existence of the resulting causality. Third, the dependence of the FFQ method on memory or cognitive performance in this study did not allow for a precise estimation of nutrient intake. Fourth, we controlled for the effects of nutrient dietary intake in the model using the residual method and VIF. Fifth, we were unable to adjust for genetically related lipoprotein E status, but we controlled for a wide range of health-related factors as potential confounders. Sixth, because we conducted a large cross-sectional epidemiological survey, the MMSE questionnaire is not clinically adequate to accurately classify cognitive impairment, and our data do not adequately support studies with a detailed classification of patients with cognitive impairment. The MMSE questionnaire can only be used to initially assess the status of cognitive function. Therefore, to obtain more precise findings, longer follow-up and larger prospective studies are necessary to explore the relationship between dietary patterns and cognitive function.

## 5. Conclusion

In this population-based study, the vegetable and mushroom dietary pattern showed a strong correlation with cognitive function, and this association was dose-dependent. Therefore, the vegetable and mushroom dietary pattern is the safest choice at this time. Finally, the vegetable and mushroom dietary pattern may be a protective factor for cognitive function, suggesting that more vegetables and mushroom foods should be consumed in the diet to prevent cognitive impairment.

## Data availability statement

The original contributions presented in the study are included in the article/Supplementary material, further inquiries can be directed to the corresponding authors.

## Ethics statement

The studies involving human participants were reviewed and approved by the Ethics Committee of Guilin Medical College. The patients/participants provided their written informed consent to participate in this study.

## Author contributions

RG and ZZ had full access to all of the data in the study, took responsibility for the integrity of the data, and the accuracy of the data analysis. RG drafted the manuscript and performed the statistical analysis. WP, JC, TL, KH, SX, and XT critically revised the manuscript for important intellectual content. YL obtained funding. YL and JQ supervised the study. All authors contributed to the article and approved the submitted version.
