# Supplementary material for: Correlation between dietary patterns and cognitive function in older Chinese adults: A representative cross-sectional study
Source: Front Nutr. 2023 Apr 4;10:1093456. doi: 10.3389/fnut.2023.1093456 (PMC10110985; doi:10.3389/fnut.2023.1093456)
Supplement: Supplementary file 1 [file Data_Sheet_1.pdf]

Supplementary Material

Correlation between dietary patterns and cognitive function in older Chinese adults: A representative cross-sectional study

Ruoyu Gou[†, a,b], You Li[†,a,b], Weiyi Pang[a,b], Jiansheng Cai[c] ,Tingyu Luo[a,b], Kailian He[a,b] , Song Xiao[a,b] , Xu Tang[c], Jian Qin[c,*] , Zhiyong Zhang[a,b,*]

[a] Department of Environmental Health and Occupational Medicine, School of Public Health, Guilin Medical University, Zhiyuan Road No.1, Guilin, 541199, Guangxi province, PR China

[b] The Guangxi Key Laboratory of Environmental Exposomics and Entire Lifecycle Heath

[c] Department of Environmental and Occupational Health, School of Public Health, Guangxi Medical University, Shuangyong Road No.22, Nanning, 530021, Guangxi province, PR China

*** Correspondence:**
rpazz@163.com (Z. Zhang);
qinjian@gxmu.edu.cn (J. Qin).
[†] These authors contributed equally to this work and share first authorship.

1 Supplementary Figures and Tables

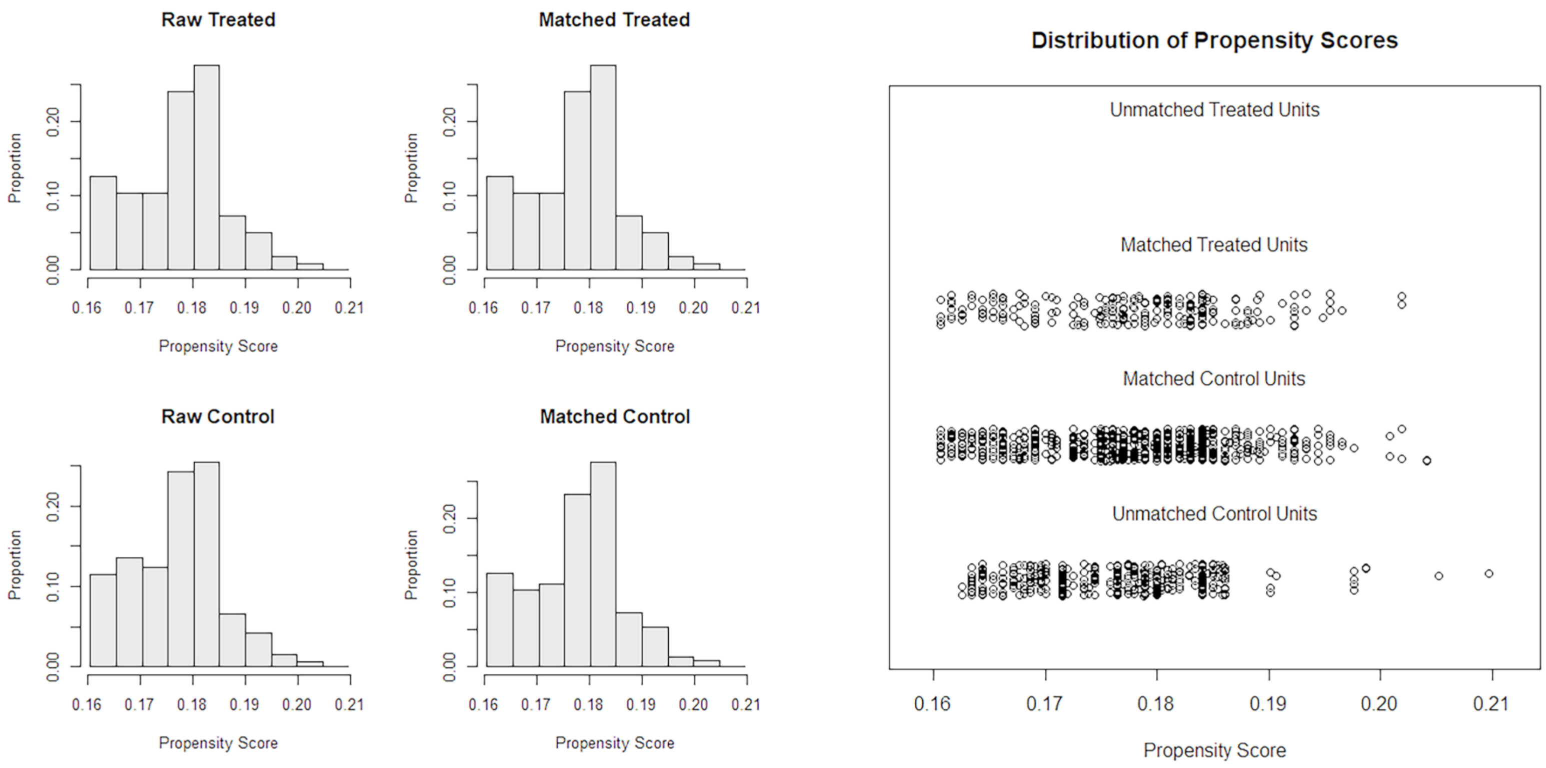

Figure 1. The left panel shows the histogram of the distribution of propensity values for propensity matching scores, and the right panel shows the distribution of propensity values for propensity matching score case dithering scatter plots.

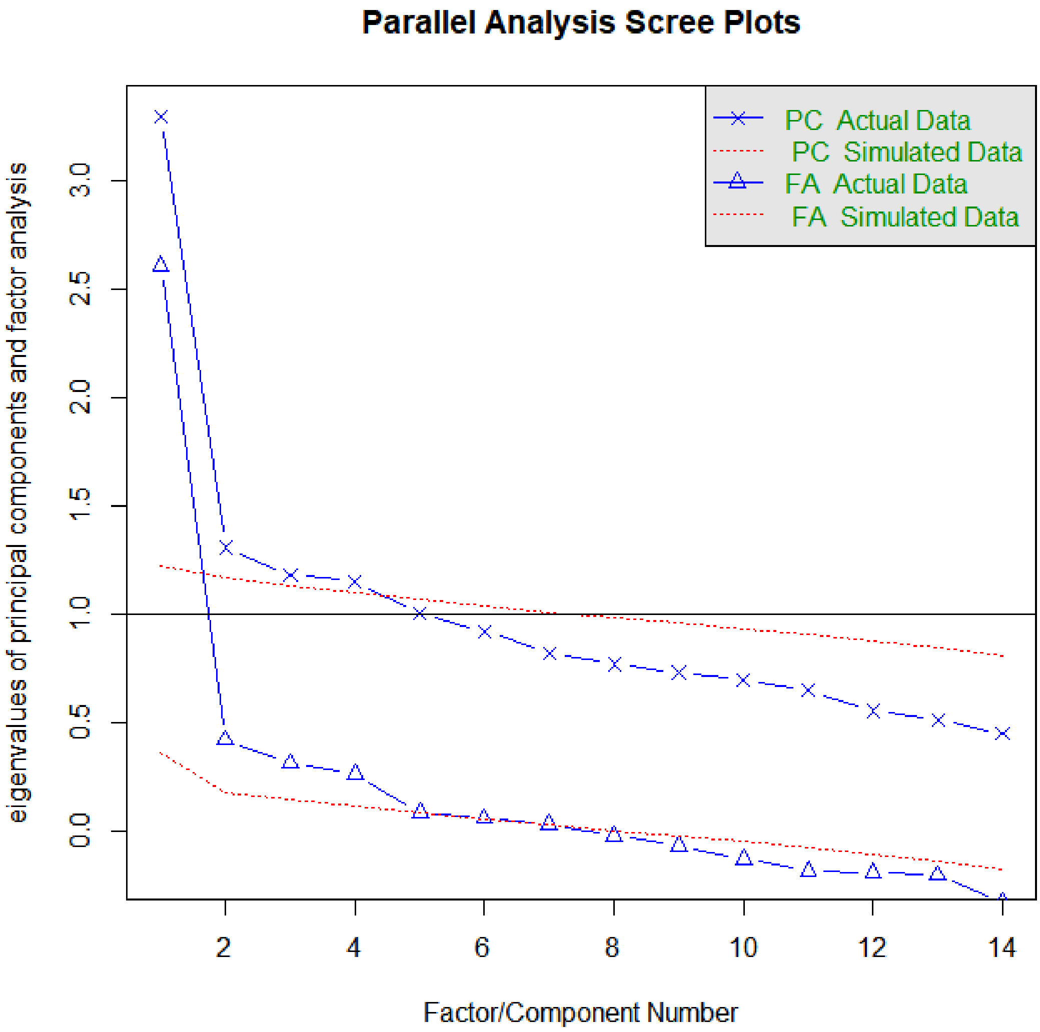

Figure 2. The scree plot of factor analysis.

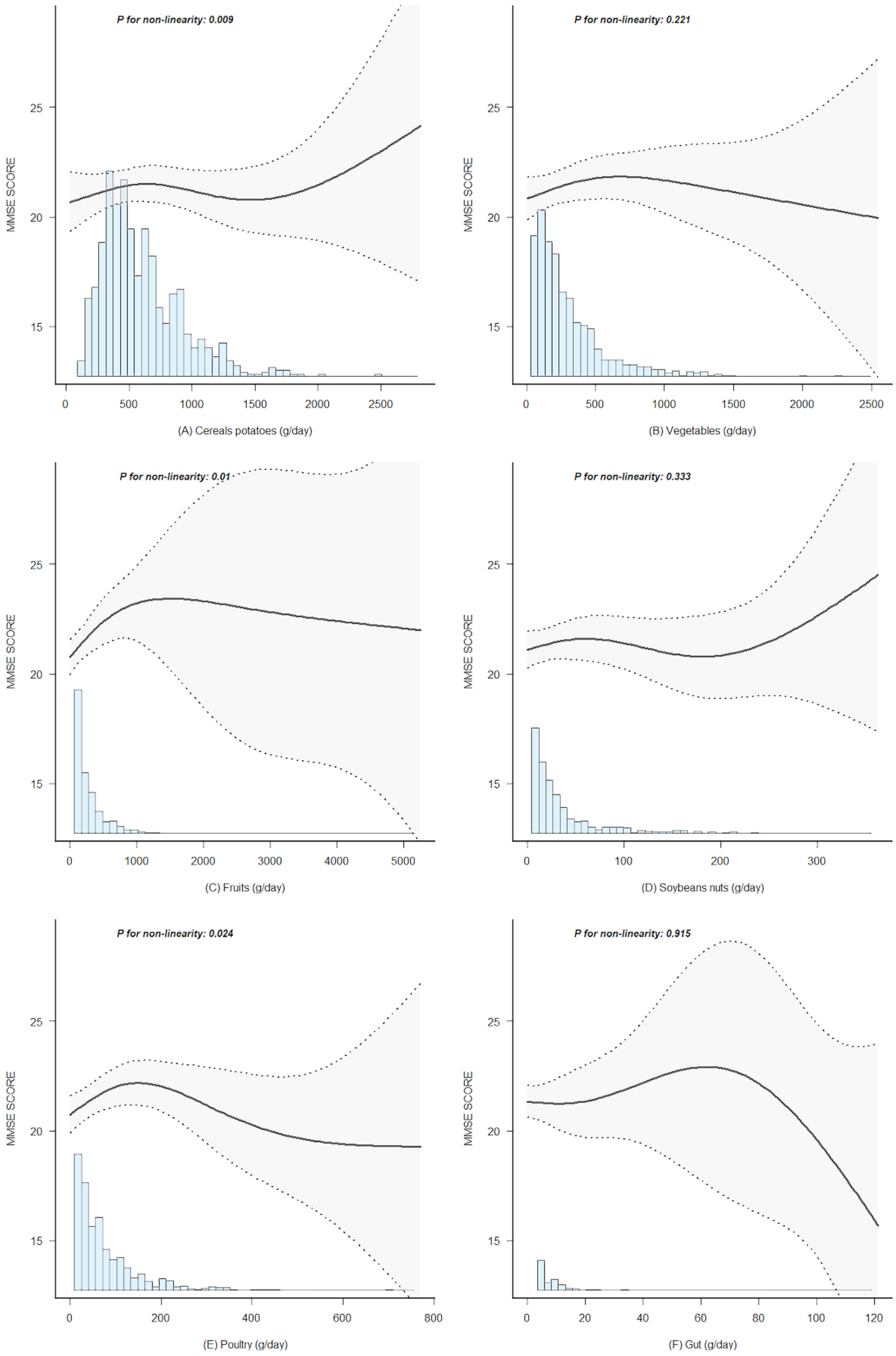

Figure 3-1. Linear trend analysis of dietary food groups and cognitive function. The logistic regression is adjusted by the following potential confounding factors: Sex, Ethnic, Marital, Agricultural activities, Smoking, Drinking, Hypertension, Diabetes, Dyslipidemia, BMI, Dietary fiber.

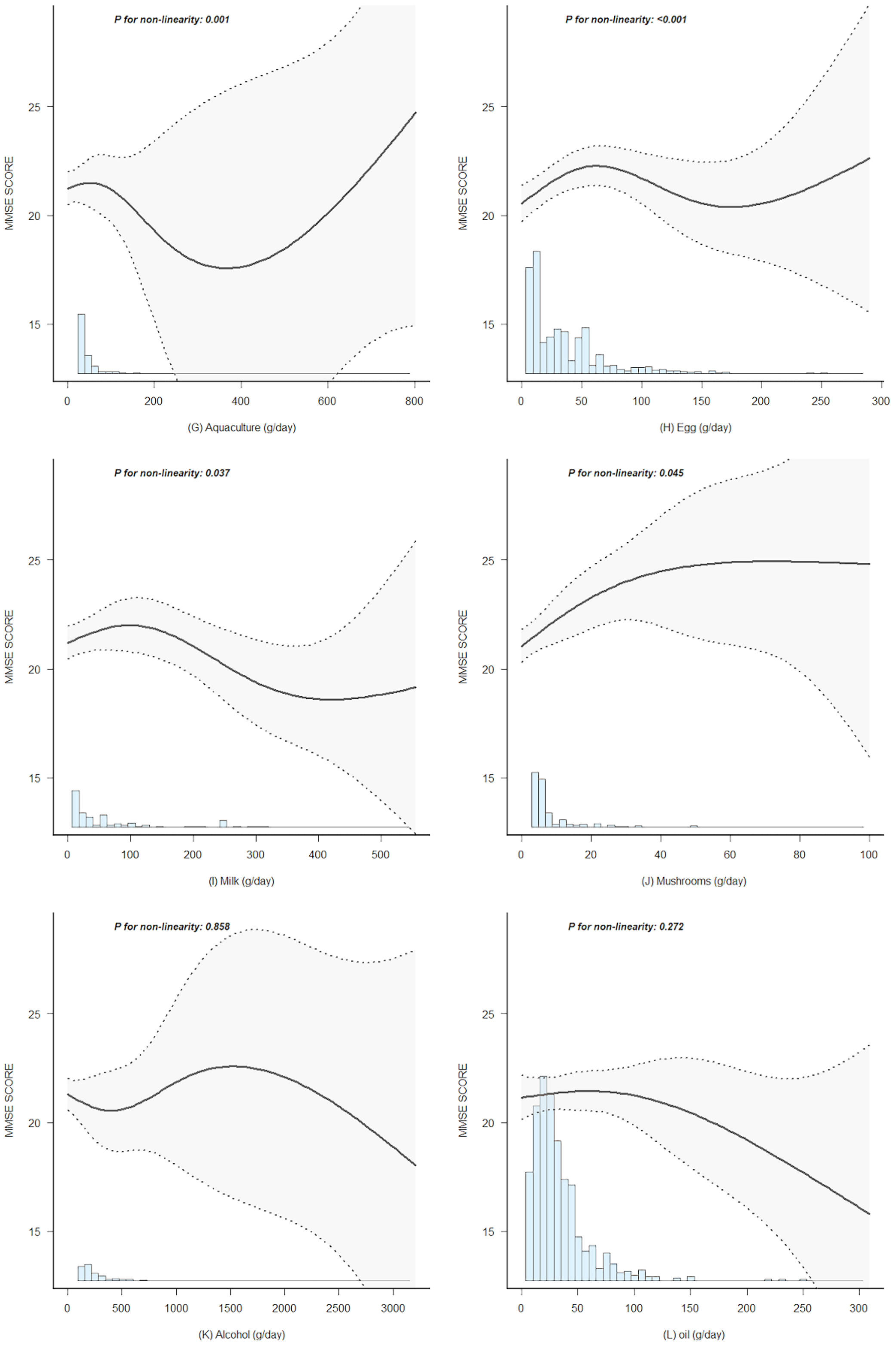

Figure 3-2. Linear trend analysis of dietary food groups and cognitive function. The logistic regression is adjusted by the following potential confounding factors: Sex, Ethnic, Marital, Agricultural activities, Smoking, Drinking, Hypertension, Diabetes, Dyslipidemia, BMI, Dietary fiber.

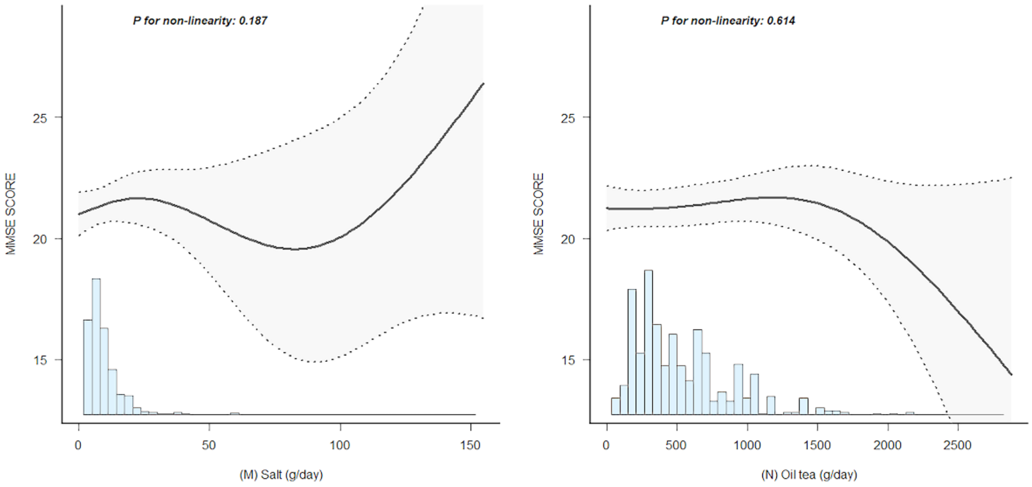

Figure 3-3. Linear trend analysis of dietary food groups and cognitive function. The logistic regression is adjusted by the following potential confounding factors: Sex, Ethnic, Marital, Agricultural activities, Smoking, Drinking, Hypertension, Diabetes, Dyslipidemia, BMI, Dietary fiber.

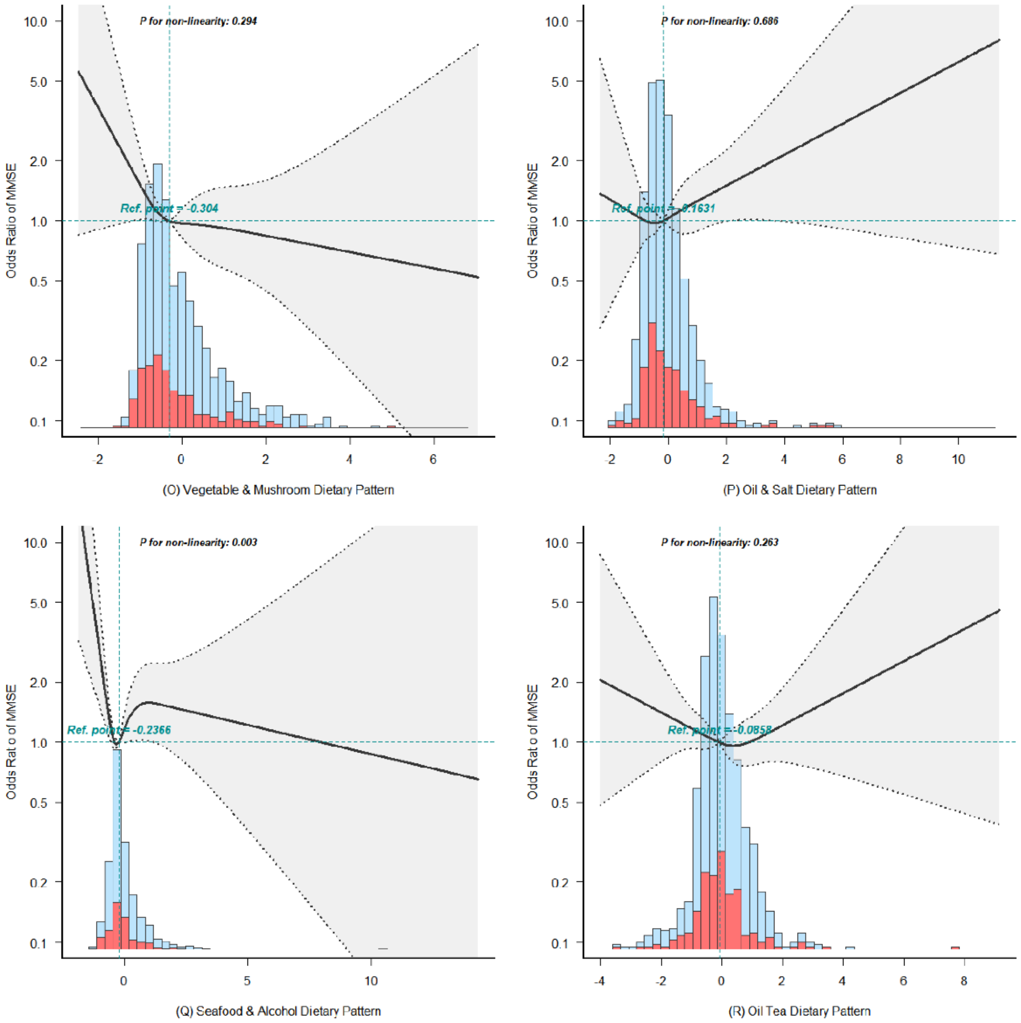

Figure 4. Restricted cubic spline plot of dietary pattern factor scores and cognitive impairment. The logistic regression is adjusted by the following potential confounding factors: Sex, Ethnic, Marital, Agricultural activities, Smoking, Drinking, Hypertension, Diabetes, Dyslipidemia, BMI, Dietary fiber.
